#  Mortality Among Severely Injured Adolescents Admitted to Pediatric vs Adult Trauma Centers

**DOI:** 10.1001/jamanetworkopen.2024.50647

**Published:** 2024-12-12

**Authors:** Fiona Muttalib, Bourke Tillmann, Genevieve Ernst, Srinivas Murthy, Zulfiqar Bhutta, Bettina Hansen, Neill K. J. Adhikari

**Affiliations:** 1 Division of Critical Care Medicine, Department of Pediatrics, University of British Columbia, Vancouver, British Columbia, Canada; 2 Interdepartmental Division of Critical Care Medicine, University of Toronto, Toronto, Ontario, Canada; 3 Division of Respirology and Critical Care Medicine, Department of Medicine, University Health Network, Toronto, Ontario, Canada; 4 Division of Emergency Medicine, Department of Pediatrics, BC Children’s Hospital, Vancouver, British Columbia, Canada; 5 Center for Excellence in Women and Child Health, Aga Khan University, Karachi, Pakistan; 6 Centre for Global Child Health, Hospital for Sick Children, Toronto, Ontario, Canada; 7 Institute of Health Policy, Management and Evaluation, Dalla Lana School of Public Health, University of Toronto, Toronto, Ontario, Canada; 8 Toronto General Hospital Research Institute (TGHRI), Toronto, Ontario, Canada; 9 Department of Critical Care Medicine, Sunnybrook Health Sciences Centre, Toronto, Ontario, Canada

## Abstract

**Importance:**

Care in a pediatric (vs adult) trauma center improves outcomes for injured children aged 0 to 12 years, but whether pediatric care benefits injured adolescents is unclear.

**Objective:**

To evaluate the association of pediatric vs adult trauma center care with mortality among severely injured adolescents.

**Design, Setting, and Participants:**

This retrospective cohort study was conducted between April 1, 2012, and March 31, 2020, among adolescents aged 12 to 16 years who were admitted to level I or level II adult trauma centers or a level I pediatric trauma center in British Columbia, Canada. Analysis was conducted between January and September 2024.

**Exposure:**

Admission to a level I pediatric trauma center or level I or level II adult trauma center.

**Main Outcomes and Measures:**

The primary outcome was hospital mortality for the index trauma incident. Inverse probability of treatment weighting was used to estimate the association of admission to a pediatric trauma center with mortality.

**Results:**

A total of 416 patients aged 12 to 16 years (median [IQR] age, 15 [13-16] years; 308 male [74.0%]) were admitted to a level I or level II trauma center with severe injury (201 [48.6%] at a pediatric trauma center; 83 [20.0%] at a level I adult trauma center; and 132 [31.7%] at a level II adult trauma center). Patients admitted to the pediatric trauma center (vs level I or level II adult centers) had lower median (IQR) age (14 [13-15] years vs 15 [14-16] years), higher median (IQR) Injury Severity Score (16 [9-21] vs 13 [9-18]) and fewer penetrating injuries (10 injuries [5.0%] vs 28 injuries [13.0%]). Hospital mortality was 7.0% (14 of 201 patients) among patients admitted to the pediatric center vs 4.2% (9 of 215 patients) among those admitted to an adult trauma center. There was no statistically significant difference in hospital mortality between patients admitted to pediatric vs adult trauma centers (adjusted odds ratio, 2.61; 95% CI, 0.88-7.69; *P* = .08).

**Conclusions and Relevance:**

In this cohort study of severely injured adolescents, pediatric trauma center admission was not associated with improved hospital mortality. These findings suggest that severely injured adolescents aged 12 to 16 years may be safely treated at either adult or pediatric trauma centers.

## Key Points

**Question:**

Is admission to a pediatric trauma center (vs adult trauma center) associated with improved survival for severely injured adolescents aged 12 to 16 years?

**Findings:**

In this cohort study of 416 severely injured adolescents, admission to a pediatric trauma center was not associated with improved hospital mortality compared with admission to an adult trauma center.

**Meaning:**

These findings suggest that severely injured adolescents aged 12 to 16 years may be safely treated at either adult or pediatric trauma centers.

## Teaser

This cohort study analyzes associations of pediatric vs adult trauma center care with mortality among adolescents with severe injury in Canada.

## Introduction

Injuries are a leading cause of mortality among children, adolescents, and young adults.^1^ To reduce mortality and morbidity, high-quality prehospital care and prompt access to specialized health care services are needed. Robust evidence shows that organized trauma systems reduce mortality and morbidity,^2^ and prior studies demonstrate that treatment at a pediatric trauma center improves survival among children aged 0 to 12 years.^3,4,5^ However, studies are conflicting for adolescents aged 12 to 18 years,^3,6,7,8,9,10,11^ who are closer in age to adults and may benefit from treatment in a higher volume center,^12,13^ particularly if need for interfacility transfer to a dedicated pediatric trauma center causes delays to reaching definitive care.^14^ In a recent systematic review, Moore and colleagues^3^ found that admission to pediatric trauma centers was associated with lower odds of mortality compared with adult trauma centers (odds ratio [OR], 0.59; 95% CI, 0.46-0.76; 34 studies). However, there were no significant differences in mortality in subgroup analyses of children 14 years or older (OR, 0.71; 95% CI, 0.47-1.06), children with an Injury Severity Score (ISS) of 12 or greater (OR, 0.60; 95%CI, 0.16-2.25), or after adjusting for interhospital transfer status (OR, 0.70; 95% CI, 0.40-1.22).^3^ Guidance related to the age threshold for admission to pediatric trauma centers is lacking, contributing to wide practice variation.^3,15^

An important limitation of current evidence is the possibility of unmeasured confounding impacting both the choice of trauma center and outcome. Prior studies have described a tendency to admit adolescents with more severe types of injury (eg, penetrating vs blunt) and higher ISS to adult trauma centers,^8,10,16^ which may mask a benefit of care in an adult trauma center. The aim of this study was to determine the association of admission to a pediatric vs adult trauma center with hospital outcomes among severely injured adolescents (12-16 years) in British Columbia (BC), Canada, using robust methods to address confounding.

## Methods

The primary objective of this cohort study was to evaluate the association of pediatric trauma center admission with hospital mortality among severely injured adolescents. The secondary objective was to evaluate the association of pediatric trauma center admission with hospital-free days at day 90. This study was approved by the University of British Columbia research ethics board. Participant consent was not required for this study because it involved secondary analysis of deidentified data in an existing dataset. This report adheres to the Strengthening the Reporting of Observational Studies in Epidemiology (STROBE) reporting guideline for cohort studies. Additional description of study methods is provided in the eMethods and eFigure 1 in Supplement 1.

### Setting

In BC, with an area of 944 735 km^2^ and seasonal extreme weather conditions, trauma care is delivered through a regional trauma system. Specialized health care services and level I trauma centers are concentrated in urban areas; however, the majority of injuries and injury-related deaths occur in rural and remote locations.^17^ There are 2 level I adult trauma centers, 4 level II adult trauma centers, and 1 level I pediatric trauma center.^18^ Children (0-16 years) who require hospitalization due to severe injury are referred preferentially to the pediatric trauma center after initial stabilization at the nearest regional trauma center.^18^

### Data Source

The study included all patients recorded in the BC Trauma Registry from April 1, 2012, to March 31, 2020. Data were obtained from Trauma Services BC and included linked data from BC Emergency Health Services, BC Vital Statistics Agency, BC Trauma Registry, BC Discharge Abstract Database, and the National Ambulatory Care Reporting System. Patients with a principal admission or discharge diagnosis of injury were identified by *International Statistical Classification of Diseases and Related Health Problems, Tenth Revision (ICD-10) *codes. Eligibility criteria for inclusion in the BC Trauma Registry are described in eTable 1 in Supplement 1.

### Study Population

We planned a priori to include all severely injured patients aged 12 to 24 years admitted to a level I or level II trauma center. In post hoc revision, in which the main analysis was shifted to propensity weighting rather than an instrumental variable approach, the primary study cohort was revised to include only patients aged 12 to 16 years. Patients were considered to have severe injury if they met any of the following criteria: ISS greater than 15, death within 24 hours of hospital arrival, or diagnosis of a life-threatening injury (eTable 2 in Supplement 1). Diagnoses considered as potentially life-threatening included uncontrolled hemorrhage; open chest wounds; penetrating trauma of the head, neck, or extremity; abdominal trauma requiring surgical intervention; amputation; fracture of 2 or more long bones; vertebral fractures; open fractures; any use of mechanical ventilation; and traumatic brain injury requiring neurosurgical assessment or intervention. We excluded patients residing outside of BC and patients with environmental injuries (eg, burns) who required subspecialty care in addition to a coordinated trauma system response.

### Patient Characteristics

We characterized patients by age, sex, median neighborhood household income level, type of hospital of initial presentation, Glasgow Coma Scale (GCS), systolic blood pressure, and heart rate at the ultimate admitting trauma center, and use of mechanical ventilation during the hospital stay (eTable 3 in Supplement 1). Only biological sex was recorded in the BC Trauma Registry. Injuries were characterized by type (blunt, penetrating, or other) and ISS (2008 version). ISS, which has a range from 1 to 75, was categorized into 4 groups (1-8, 9-15, 16-24, and 25-75).^19^ Patients who died within 24 hours of admission were considered to have the highest category of ISS. Euclidean distance from the incident latitude and longitude recorded in the discharge abstract database to the nearest level I or level II trauma center and to the trauma center to which the patient was ultimately admitted were estimated using the Geosphere package in R Studio version 1.2.1335 (Posit).^20^ Median neighborhood household income level was estimated using 2016 Canada census data by forward sortation area (first 3 alphanumeric characters of the home address postal code).^21^

### Exposure

The main exposure was the type of trauma center to which the patient was ultimately admitted for the index trauma admission (referred to hereafter as admission hospital). Exposure was dichotomized as either a pediatric or adult trauma center, as recorded in the BC Trauma Registry.

### Outcomes

The primary outcome was hospital mortality for the index trauma incident. The secondary outcome was hospital-free days at 90 days for the index hospital admission. Patients who died within 90 days or had hospital length of stay greater than 90 days were assigned 0 hospital-free days.

### Statistical Analysis

Study cohort characteristics were summarized descriptively for the overall cohort and stratified by admitting facility. All statistical analyses were performed using R Studio and Stata/SE version 17.0 for Mac (StataCorp).^22,23^ Statistical significance was considered as a 2-sided *P* < .05. Data analysis occurred from January to September 2024.

#### Missing Data

Missing data were summarized descriptively (eTable 4 in Supplement 1). For participants for whom trauma event location was missing, the centroid longitude and latitude of the forward sortation area of the home address was used as the event location. Where accepting facility GCS was missing, scene GCS was used, if available. For remaining missing data, multiple imputation by chained equations with 5 imputed datasets was used for the primary analysis.

#### Propensity Score Analysis

In post hoc revision, the primary analysis was conducted using a logistic regression model with inverse probability of treatment weighting. We first handled missing data as specified previously. In each imputed dataset, we estimated the probability of admission to pediatric trauma center among adolescents aged 12 to 16 years, given prespecified covariates (sex, ISS, presence of systolic blood pressure <90 mm Hg, presence of GCS <9, any use of mechanical ventilation, injury type, and time to reaching the admitting hospital). We constructed average treatment effect weights and verified the balance in measured covariates between the exposed patients (admitted to the pediatric trauma center) and unexposed patients (admitted to the level I or level II adult trauma center) using standardized differences (eTable 5 in Supplement 1).^24^ We estimated the average treatment effect of admission to the pediatric trauma center within each dataset, using logistic regression with inverse probability of treatment weighting, and then pooled estimates.^24^ Clinical data for adjustment were obtained at the admission hospital. Robust sandwich estimators were used to calculate standard errors to account for the risk of heteroskedasticity associated with binary outcome variables.^25^

#### Secondary Analysis and Sensitivity Analyses

As a secondary analysis, we evaluated the association of admission to the pediatric trauma center with hospital-free days using a negative binomial regression model. We conducted sensitivity analyses with different subpopulations including patients with blunt injuries, patients with penetrating injuries, and transferred patients. In addition, we used instrumental variable analysis to evaluate the average treatment effect of admission to a pediatric trauma center in a cohort of severely injured adolescents and adults aged 12 to 24 years (see eMethods in Supplement 1). Age category was used as an instrument for admission to a pediatric vs adult trauma center to estimate the average population treatment effect of admission to a pediatric trauma center due to threshold age less than 17 years on hospital mortality.

## Results

From April 1, 2012, to March 31, 2020, 416 patients aged 12 to 16 years (median [IQR] age, 15 [13-16] years; 308 male [74.0%]) were admitted to a level I or level II trauma center with severe injury (eFigure 2 and eFigure 3 in Supplement 1 and Table 1). No patients were excluded due to missing age or outcome data (eFigure 2 and eTable 4 in Supplement 1). Nearly one-half were admitted to the pediatric trauma center (201 participants [48.6%]), 132 were admitted to a level II adult trauma center (31.7%), and 83 were admitted to a level I adult trauma center (20.0%). Patients admitted to the pediatric trauma center had lower median (IQR) age (14 [13-15] vs 15 [14-16] years), higher median (IQR) ISS (16 [9-21] vs 13 [9-18]), and fewer penetrating injuries (10 injuries [5.0%] vs 28 injuries [13.0%]) (Table 1). Most injuries were blunt (175 injuries [87.1%] at the pediatric trauma center and 181 injuries [84.2%] at adult trauma centers). A lower proportion of patients admitted to the pediatric trauma center were direct admissions compared with patients admitted to adult trauma centers (73 patients [36.3%] vs 144 patients [67.0%]). Patients admitted to the pediatric trauma center had incidents further from any level I or level II trauma center (median [IQR] distance, 23.1 [6.6-68.2] km vs 16.1 [6.7-61.4] km), further from the admission trauma center (median [IQR] distance, 39.3 [9.5-98.2] km vs 18.2 [6.9-75.1] km), and had longer time to arrival at the admission trauma center (median [IQR] time, 4.7 [1.5-8.3] hours vs 1.6 [0.7-5.4] hours). Eighteen patients were referred from a level I or level II adult trauma center to the pediatric trauma center.

**Table 1.** Characteristics of Patients Aged 12 to 16 Years Admitted to PTCs vs ATCs

Variable	Participants, No. (%)			ATC vs PTC, standardized difference
	Overall (N = 416)	Level I PTC (n = 201)	Level I or II ATC (n = 215)	
Age, median (IQR), y	15 (13-16)	14 (13-15)	15 (14-16)	0.76
Sex				
Female	108 (26.0)	51 (25.4)	57 (26.5)	0.03
Male	308 (74.0)	150 (74.6)	158 (73.5)	
Injury type				
Blunt	356 (85.6)	175 (87.1)	181 (84.2)	0.38
Asphyxia	16 (3.8)	13 (6.5)	3 (1.4)	
Other or unspecified	6 (1.4)	3 (1.5)	3 (1.4)	
Penetrating	38 (9.1)	10 (5.0)	28 (13.0)	
ISS, median (IQR)	14 (9-21)	16 (9-21)	13 (9-18)	0.98
ISS category^a^				
1-8	94 (22.6)	45 (22.4)	49 (22.8)	0.27
9-15	123 (29.6)	48 (23.9)	75 (34.9)	
16-24	115 (27.6)	65 (32.3)	50 (23.3)	
25-75	84 (20.2)	43 (21.4)	41 (19.1)	
Mechanical ventilation	123 (29.6)	62 (30.8)	61(28.4)	0.05
GCS, median (IQR)	15 (14-15)	15 (14-15)	15 (14-15)	0.37
Heart rate, median (IQR), bpm	90 (76-105)	94 (80-106)	88 (76-102)	2.90
SBP, median (IQR), mm Hg	120 (113-131)	118 (110-127)	123 (115-135)	5.30
Shock index, median (IQR)	0.8 (0.6-0.9)	0.8 (0.7-0.9)	0.7 (0.6-0.9)	−0.05
SBP <90 mm Hg	16 4.1)	7 (3.8)	9 (4.3)	0.03
GCS <9, No. (%)	49 (13.6)	25 (15.2)	24 (12.2)	0.09
First facility level				
I (Adult)	75 (18.0)	13 (6.5)	62 (28.8)	1.80
I (Pediatric)	75 (18.0)	73 (36.3)	2 (0.9)	
II	90 (21.6)	5 (2.5)	85 (39.5)	
III	27 (6.5)	22 (10.9)	5 (2.3)	
IV	29 (7.0)	23 (11.4)	6 (2.8)	
Other	120 (28.8)	65 (32.3)	55 (25.6)	
Direct admission	217 (52.2)	73 (36.3)	144 (67.0)	0.64
Accepting facility level				
I (Adult)	83 (20.0)	0	83 (38.6)	0.33
I (Pediatric)	201 (48.3)	201 (100.0)	0	
II	132 (31.7)	0	132 (61.4)	
Minimum distance to level I or II TC, median (IQR), km	20.4 (6.6-67.8)	23.1 (6.6-68.2)	16.1 (6.7-61.4)	42.00
Distance to the accepting TC, median (IQR), km	26.4 (8.4-90.5)	39.3 (9.5-98.2)	18.2 (6.9-75.1)	62.00
Time to the accepting facility, median (IQR), h	3.0 (0.9-7.3)	4.7 (1.5-8.3)	1.6 (0.7-5.4)	1.60
Nearest TC level				
I (Adult)	72 (17.3)	53 (26.4)	19 (8.9)	0.71
I (Pediatric)	54 (13.0)	40 (19.9)	14 (6.5)	
II	289 (69.6)	108 (53.7)	181 (84.6)	
Median neighborhood household income, median (IQR), CAD$^b^	70 313 (62 756-81 713)	70 212 (62 756-82 530)	70 743 (62 344-79 829)	546.00

Abbreviations: ATC, adult trauma center; bpm, beats per minute; GCS, Glasgow Coma Scale; ISS, injury severity score; PTC, pediatric trauma center; SBP, systolic blood pressure; TC, Trauma center.

^a^ Patients who died within 24 hours of admission are assigned the highest ISS category.

^b^ To convert to US dollars, multiply by 0.7169.

### Association of Type of Trauma Center With Outcomes

Crude hospital mortality was 7.0% (14 of 201 patients) at the pediatric trauma center and 4.2% (9 of 215 patients) at level I and level II adult trauma centers (Table 2). Patients admitted to the pediatric trauma center had similar hospital-free days at 90 days compared with patients admitted to level I or level II adult trauma centers (median [IQR] time, 86 [82-88] days vs 86 [81-88] days).

**Table 2.** Hospital Outcomes of Adolescents Hospitalized Due to Severe Injury

Outcome	Participants, median (IQR)		
	Overall (N = 416)	Level I pediatric trauma center (n = 201)	Level I or II adult trauma center (n = 215)
Hospital-free days at 90 d	86 (82-88)	86 (82-88)	86 (81-88)
Length of stay	4 (2-8)	4 (2-8)	4 (2-9)
Death, No. (%)	23 (5.5)	14 (7.0)	9 (4.2)

Using inverse probability treatment weighting logistic regression, there was no statistically significant association of admission to the pediatric trauma center with hospital death (adjusted OR, 2.61; 95% CI, 0.88-7.69; *P* = .08) (eTable 6 in Supplement 1). There was no association of admission to the pediatric trauma center with hospital-free days at day 90 (incidence rate ratio, 1.02; 95% CI, 0.99-1.06; *P* = .20) (eTable 7 in Supplement 1). In prespecified sensitivity analyses using different population subgroups defined by injury type and transfer status, the point estimate of the average treatment effect of admission to a pediatric trauma center was comparable to the primary analysis. In a larger cohort of severely injured adolescents and adults 12 to 24 years (eFigure 3, eResults, and eTable 8 in Supplement 1), after adjusting for differences in baseline characteristics, instrumental variable analysis demonstrated that admission to pediatric trauma center was associated with an average 5% increased absolute risk of hospital mortality (risk difference, 0.05; 95% CI, 0.00-0.09; *P* = .04) (eTable 9 and eTable 10 in Supplement 1).

## Discussion

In this cohort study of severely injured adolescents, pediatric trauma center (vs adult) was not associated with improved hospital mortality in the primary propensity weighting analysis. Our findings suggest that adolescents 12 to 16 years with severe injury may be safely treated at adult and pediatric trauma centers.

The study findings are consistent with existing US literature that has demonstrated similar hospital mortality for injured adolescents admitted to adult and pediatric trauma centers.^3,8,9,10,16^ Although our results conflict with a recent US study^4^ that included a subgroup analysis of 405 adolescents with severe injury (ISS >15), demonstrating a mortality advantage of admission to pediatric trauma centers (OR, 0.70; 95% CI, 0.53-0.91), the comparison group combined admission to adult trauma centers and non–trauma centers. Of note, our sensitivity analysis using an instrumental variable suggested increased mortality in the pediatric trauma center.

An important difference between the Canadian and US context is the nearly 2-fold availability of pediatric trauma centers per capita in the US, with a greater proportion of the US population living within 1 hour of a level I pediatric trauma center.^6^ Therefore, geographic factors may play a lesser role in the impact of pediatric trauma center admission or transfer in the US compared with Canada. In our study, patients admitted to the pediatric trauma center traveled further and took longer to reach the admission facility. Sensitivity analysis including only patients who underwent interfacility transfer demonstrated similar results to the primary analysis; however, this analysis was limited by small sample size. Further research is needed to identify factors, including transport characteristics, which may modify the association of trauma center type with hospital mortality.

### Limitations

There are several limitations to consider in the interpretation of study results. First, the study draws upon exclusively observational retrospective data voluntarily entered into the BC Trauma Registry. Trauma patients who died before transfer to a BC Trauma Registry center would not be included; however, this phenomenon would not be expected to be differential by site of admission. Second, the study sample size was small with few deaths. The small sample size may contribute to overestimation of robust standard errors of the association of pediatric trauma center admission with mortality.^26^ Third, ISS is limited as a severity of injury measure because it cannot account for multiple injuries to a given body region and does not describe severity of physiologic derangement. For this reason, we included other validated measures of illness severity such as systolic blood pressure, GCS, and any use of mechanical ventilation in the propensity score analysis. However, we were limited by measurement of these variables at the admitting hospital, and the time of initiation of mechanical ventilation was unknown. Future studies may benefit from use of a physiology-based mortality prediction score for risk adjustment.^27^ Fourth, there may be important short-term and long-term nonmortality benefits to hospitalization at a dedicated pediatric trauma center for the majority of patients who survive. In this study, we were not able to examine long-term physical or psychological functional status, health-related quality of life, family consequences, and health care utilization after discharge,^28^ which may all be better at pediatric trauma centers that provide family-centered care and specialized pediatric psychological supports.

## Conclusions

In this cohort study of severely injured adolescents, there was no association of trauma center type (adult vs pediatric) with hospital mortality. Where appropriate for resource management and to reduce time to definitive care, it may be safe to treat adolescents aged 12 to 16 years at level I and level II adult trauma centers. Further research is needed to determine whether the findings generalize to geographically large trauma systems in Canada and other countries.

## Notes

Supplement 1. **eMethods. **Instrumental Variable Analysis **eFigure 1.** Directed Acyclic Graph of the Association between Pediatric Trauma Centre Admission and Mortality **eTable 1.** BC Trauma Registry Inclusion and Exclusion Criteria **eTable 2.** Criteria for Severe Injury **eTable 3.** Study Variable List **eTable 4.** Frequency of Missing Data in Analytic Sample **eTable 5.** Balance Summary Across All Imputations **eFigure 2.** Patients 12–16 Years Included in the Primary Analysis **eTable 6.** Association Between Trauma Centre Type and Mortality Using Logistic Regression With Inverse Probability Treatment Weighting **eTable 7.** Multivariable Model for Association Between Trauma Centre Type and Hospital-Free Days at 90 Days **eFigure 3.** Patients 12–24 Years Included in the Instrumental Variable Analysis
eResults.
**eTable 8.** Characteristics of Adolescents and Young Adults 12–24 Years Hospitalized due to Severe Injury **eTable 9.** Multivariable Model for Association Between Trauma Centre Type and Mortality Among Severely Injured Adolescents and Adults Using Instrumental Variable Regression **eTable 10.** Comparison of IV Estimation Methods of the Average Effect of PTC Admission on Hospital Mortality
eReferences

Supplement 2.
Data Sharing Statement
