# Supplementary material for: Mortality Among Severely Injured Adolescents Admitted to Pediatric vs Adult Trauma Centers
Source: JAMA Netw Open. 2024 Dec 12;7(12):e2450647. doi: 10.1001/jamanetworkopen.2024.50647 (PMC11638793; doi:10.1001/jamanetworkopen.2024.50647)
Supplement: Supplement 1. — eMethods. Instrumental Variable Analysis eFigure 1. Directed Acyclic Graph of the Association between Pediatric Trauma Centre Admission and Mortality eTable 1. BC Trauma Registry Inclusion and Exclusion Criteria eTable 2. Criteria for Severe Injury eTable 3. Study Variable List eTable 4. Frequency of Missing Data in Analytic Sample eTable 5. Balance Summary Across All Imputations eFigure 2. Patients 12–16 Years Included in the Primary Analysis eTable 6. Association Between Trauma Centre Type and Mortality Using Logistic Regression With Inverse Probability Treatment Weighting eTable 7. Multivariable Model for Association Between Trauma Centre Type and Hospital-Free Days at 90 Days eFigure 3. Patients 12–24 Years Included in the Instrumental Variable Analysis eResults. eTable 8. Characteristics of Adolescents and Young Adults 12–24 Years Hospitalized due to Severe Injury eTable 9. Multivariable Model for Association Between Trauma Centre Type and Mortality Among Severely Injured Adolescents and Adults Using Instrumental Variable Regression eTable 10. Comparison of IV Estimation Methods of the Average Effect of PTC Admission on Hospital Mortality eReferences [file jamanetwopen-e2450647-s001.pdf]

Supplemental Online Content

Muttalib F, Tillmann B, Ernst G, et al. Mortality among severely injured adolescents admitted to pediatric vs adult trauma centers. JAMA Netw. Open. 2024;7(12):e2450647. doi:10.1001/jamanetworkopen.2024.50647

eMethods. Instrumental Variable Analysis
eFigure 1. Directed Acyclic Graph of the Association between Pediatric Trauma Centre Admission and Mortality
eTable 1. BC Trauma Registry Inclusion and Exclusion Criteria
eTable 2. Criteria for Severe Injury
eTable 3. Study Variable List
eTable 4. Frequency of Missing Data in Analytic Sample
eTable 5. Balance Summary Across All Imputations
eFigure 2. Patients 12–16 Years Included in the Primary Analysis
eTable 6. Association Between Trauma Centre Type and Mortality Using Logistic Regression With Inverse Probability Treatment Weighting
eTable 7. Multivariable Model for Association Between Trauma Centre Type and Hospital-Free Days at 90 Days
eFigure 3. Patients 12–24 Years Included in the Instrumental Variable Analysis
eResults
eTable 8. Characteristics of Adolescents and Young Adults 12–24 Years Hospitalized due to Severe Injury
eTable 9. Multivariable Model for Association Between Trauma Centre Type and Mortality Among Severely Injured Adolescents and Adults Using Instrumental Variable Regression
eTable 10. Comparison of IV Estimation Methods of the Average Effect of PTC Admission on Hospital Mortality
eReferences

This supplemental material has been provided by the authors to give readers additional information about their work.

eMethods. Instrumental Variable Analysis

Instrumental variable analysis was used to account for potential unmeasured confounders impacting site of hospital admission. The basis of our instrumental variable analysis is the assumption that there should be no baseline differences in mortality risk between 12–16 year old and 17–24 year old severely injured youth, and therefore observed differences in mortality may be related to differences in management at dedicated adult versus pediatric sites. Three assumptions must be met to ensure the validity of an instrumental variable: i) there must be a strong association between the instrumental variable and the exposure of interest, ii) there are no unmeasured common causes of the instrumental variable and the outcome of interest, and iii) there must be no association between the instrumental variable and the outcome of interest independent of treatment assignment (eFigure 1).[1] Age category is an instrumental variable for admission to PTC consistent with these assumptions. Age category is strongly associated with decision to admit or transfer to PTC, and this assumption can be verified empirically. Although assumptions ii and iii cannot be verified empirically, there was no evidence of manipulation of age in the dataset and no potential for a common cause of age category and the outcome. There is also no a priori reason to consider that there is another pathway from age category to mortality other than its impact on facility of admission. There are no other threshold changes that occur at age 16 that imply differences in risk of severe injury or management of injury. Factors associated with increased risk of mortality were compared across the two groups and important covariates for mortality were included in the regression analysis (eFigure 1). A fourth assumption has been proposed to ensure the validity of point estimates of the causal effect of the exposure on the outcome: the absence of effect modification in patient subgroups.[1] We examined this assumption by comparing the PTC vs ATC effect on mortality in patients 12–16 years old and 17–24 years old at the ATC.
The impact of admission to PTC due to age threshold was evaluated using two-stage least squares regression (2SLS).[2,3]

Equation 1 $g[E(T_i)] = \delta_0 + \delta_1 \times (Z_i) + \delta_2 \times C_i + \varepsilon_{1i}$; for i=1,2,…n

Equation 2 $g[E(Y_i)] = \beta_0 + \beta_{IV} \times Xi + \beta_2 \times C_i + \varepsilon_{2i}$; for i=1,2,…n

Where,

g: generic link function

Y_i: Rate of mortality by facility type (PTC versus ATC)

Ti: Rate of admission to PTC

Z_i : instrumental variable age category

C: confounders

β_{IV}: Estimator of the causal relationship between facility type and mortality

The impact of admission to PTC on hospital mortality was evaluated by first determining the probability of admission to PTC by age group in equation 1, accounting for relevant co-variates. This probability function was then incorporated into equation 2 to determine the mortality risk difference (β_{IV}) among eligible patients admitted to PTC, accounting for other covariates for hospital death. Robust sandwich estimators were used to calculate standard errors to account for the risk of heteroskedasticity associated with binary

© 2024 Muttalib et al. *JAMA Netw. Open*.

outcome variables.[4] We considered alternate estimation methods for the IV analysis to minimize bias of results. [2,5]

© 2024 Muttalib et al. *JAMA Netw. Open*.

eFigure 1: Directed Acyclic Graph of the Association between Pediatric Trauma Centre Admission and Mortality

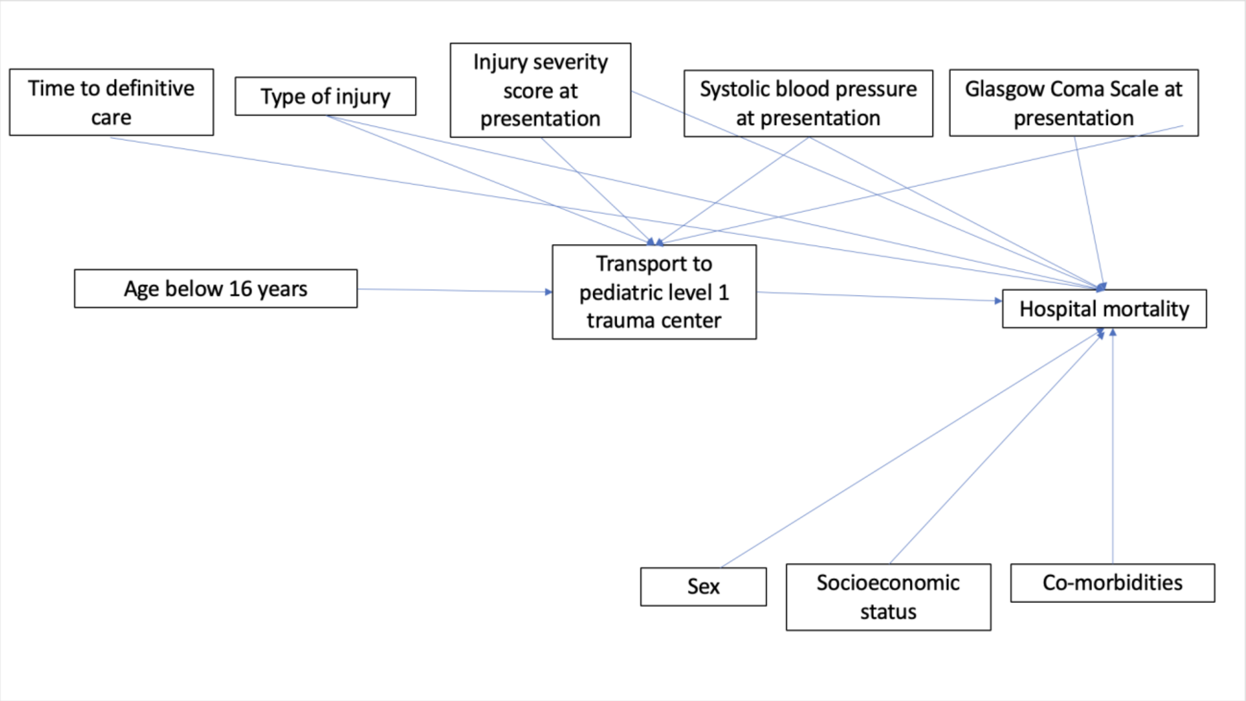

© 2024 Muttalib et al. *JAMA Netw. Open*.

eTable 1: BC Trauma Registry Inclusion and Exclusion Criteria

Inclusion Criteria
• All admitted patients who had a Trauma Team Activation (TTA)<br>• All admitted patients who met site TTA criteria regardless of any other factor.<br>• Patients who sustained injury caused by a select external cause, arrived at hospital within 21 days (or part of a continuous acute care transfer), and met one (or more) of the following criteria:<br>○ Died in ED or in hospital within 24 hours of admission, regardless of ISS.<br>○ Admitted, >15 years of age and had an ISS ≥9.<br>○ Admitted, ≤15 years of age, regardless of ISS.<br>○ Transferred out of a BCTR facility for a higher level of trauma care, regardless of admission or ISS.
Exclusion Criteria
• Daycare and Outpatient encounters.<br>• Elderly (≥65 years) patients with isolated hip fractures due to some same level falls.<br>• Psychiatric admissions for self-inflicted injuries where admission is for underlying psychiatric disorder rather than for injuries sustained.<br>• Pathological fractures with no mechanism of injury (i.e. spontaneous fractures).<br>• Cellulitis / infection / abscess arising as complications of lacerations, animal bites, etc.<br>• Poisonings / overdoses.<br>• Decompression sickness.<br>• Fractures that are old or indeterminate if a fall occurred.<br>• Foreign body in hollow viscus (esophagus, rectum, etc.) with no anatomical injury.<br>• Planned readmissions within 21 days of injury with definitive trauma treatment addressed in previous admission.

© 2024 Muttalib et al. *JAMA Netw. Open*.

eTable 2: Criteria for severe injury

Any of the following:

ISS >15

Death within 24 hours

A diagnosis of severe injury, including:

- Uncontrolled hemorrhage
- Open chest wound
- Penetrating trauma (head, neck, extremity)
- Abdominal trauma requiring surgical intervention
- Amputation
- Fracture (2 or more long bones)
- Fracture vertebral
- Open fracture
- Mechanical ventilation
- Traumatic brain injury requiring neurosurgical assessment or intervention

© 2024 Muttalib et al. *JAMA Netw. Open*.

eTable 3: Study Variable List

VARIABLE	DESCRIPTION
Age	At the time of injury
Sex	Recorded sex
Residential Forward Sortation Area	Primary residence, first three digits only
Rsidential latitude	Latitude of primary residence
Residential longitude	Longitude of primary residence
Comorbidities	Recorded comorbidities
Primary cause of injury	ICD 10-CA codes
Primary injury type	Type of mechanism causing most serious injury
Mechanism of injury	Descriptor of. mechanism
incident province	Reported location of injury
Incident city	Reported location of injury
Incident postal code	Reported location of injury
Incident longitude	Reported location of injury
Incident latitude	Reported location of injury
Primary transport mode	Transport mode by ambulance, air ambulance (both helicopter and fixed wing), or private vehicle.
Arrived at scene – time	Time of EHS arrival to scene
Departure from scene – time	Time of EHS departure from scene
Scene time	Total time (minutes) at scene
Auto-launch?	Was an auto-launch activated for the patient?
Scene Glasgow Coma Scale (GCS)	GCS is a validated scale to describe level of consciousness among children and adults. It ranges from 3 (deep unconsciousness) to 15 (fully conscious).
Scene Intubation	Insertion of endotracheal tube at scene
Scene Systolic blood pressure	Measured systolic blood pressure
Scene Heart rate	Measured heart rate by cardiac monitor or pulse oximetry
Scene Oxygen saturation	Measured saturation by pulse oximetry
Facility name	Hospital or site first establishing duty of care to patient
Registration date	Date of registration at first facility
Registration time	Time of arrival at first facility
Separation date	Date of departure from first facility
Separation time	Time of departure from first faciity
Length of stay	Number of hours patient stayed at first facility before transfer
First facility Glasgow Coma Scale (GCS)	GCS is a validated scale to describe level of consciousness among children and adults. It ranges from 3 (deep unconsciousness) to 15 (fully conscious).
First facility systolic blood pressure	Measured systolic blood pressure
First facility heart rate	Measured heart rate by cardiac monitor or pulse oximetry
First facility oxygen saturation	Measured saturation by pulse oximetry

© 2024 Muttalib et al. *JAMA Netw. Open*.

First facility airway Management	The most invasive airway used within 4 hours of arrival
First facility Pediatric trauma score (PTS) – total	PTS at first facility
ICD-10-CA codes	Descriptions of injuries based on ICD-10-CA codes provided by International Classification of Diseases used for coding injuries and other medical conditions (S00-T98)
Time to accepting facility	Total time in hours to the accepting facility from the incident
Arrival destination	Location of admission at accepting facility
Signs of life	Arrived with or without signs of life
Accepting facility systolic blood pressure	Measured systolic blood pressure
Accepting facility heart rate	Measured heart rate by cardiac monitor or pulse oximetry
Accepting facility oxygen saturation	Measured saturation by pulse oximetry
Accepting facility Glasgow coma scale	GCS is a validated scale to describe level of consciousness among children and adults. It ranges from 3 (deep unconsciousness) to 15 (fully conscious).
Accepting facility airway management	The most invasive airway used within 4 hours of arrival
Accepting facility intubated	Intubation status, yes/no
Accepting facility Injury Severity Score (ISS)	Calculated measure of severity of injury ranging from 1 to 75 with 75 being the most severe13, 14
ED Length of stay	Total time, in hours, spent in the ED at the accepting facility (auto-calculated)
ED Disposition	Destination upon discharge from the ED at accepting facility
Number of Operations (OR visits)	OR visits during hospitalization for the traumatic injury
Operating Service	Which subspecialty performed the surgery
Emergency department major procedures	Which procedures were done in the emergency department before admission to ward, ICU, or operating room
Complications	Complications recorded as ICD 10 diagnosis (per Appendix 1 of BC Trauma Registry)
Length of stay in the emergency department	Number of hours the patient stayed in the ED prior to admission or transfer
Total hospital days	Number of days the patient stayed at accepting facility as an inpatient.
Total intensive care unit days	Number of days the patient stayed in ICU.
Total days ventilated	Number of days the patient was mechanically ventilated.
Total special care unit days	Number of days the patient was admitted in a special care unit
Length of stay (days)	Total time in days admitted at the accepting facility
Expired	Whether patient died in the hospital, regardless of etiology
Resuscitation attempted	Was lifesaving procedure attempted after cardiac/respiratory arrest

© 2024 Muttalib et al. *JAMA Netw. Open*.

Expired location	Location at time of death
Separation disposition	If patient was discharged home or to another facility, including name of facility.<br>1 – Acute Care Facility<br>2 – Against Medical Advice<br>13 – Psychiatric Facility<br>15 – Special Rehab Facility<br>16 – General Rehab Facility<br>17 – Home with Support Services<br>18 – Home<br>21 – Foster Care and/or Children’s Aid<br>22 – Hospital/Palliative Care<br>23 – Other*<br>24 – Expired<br>*This includes drug/alcohol detoxification centres or prison.
Separation status	Status at discharge from accepting facility

© 2024 Muttalib et al. *JAMA Netw. Open*.

eTable 4: Frequency of Missing Data in Analytic Sample

	Total (N=416)	Level 1 or 2 ATC (N=215)	Level 1 PTC (N=201)
Age, N (%)	0 (0.0)	0 (0.0)	0 (0.0)
Female sex, N (%)	0 (0.0)	0 (0.0)	0 (0.0)
Injury Type, N (%)	0 (0.0)	0 (0.0)	0 (0.0)
ISS, N (%)	0 (0.0)	0 (0.0)	0 (0.0)
Mechanical ventilation, N (%)	0 (0.0)	0 (0.0)	0 (0.0)
Accepting facility GCS, N (%)	56 (13.5)	19 (8.8)	37 (18.4)
Accepting facility motor GCS score, N (%)	74 (17.8)	22 (10.2)	52 (25.9)
Accepting facility HR, N (%)	13 (3.1)	8 (3.7)	5 (2.5)
Accepting facility SBP, N (%)	23 (5.5)	8 (3.7)	15 (7.5)
Accepting facility SI, N (%)	28 (6.7)	11 (5.1)	17 (8.5)
SBP <90, N (%)	23 (5.5)	8 (3.7)	15 (7.5)
GCS < 9, N (%)	56 (13.5)	19 (8.8)	37 (18.4)
First facility level, N (%)	0 (0.0)	0 (0.0)	0 (0.0)
Accepting facility level, N (%)	0 (0.0)	0 (0.0)	0 (0.0)
Direct admission, N (%)	0 (0.0)	0 (0.0)	0 (0.0)
Minimum distance to level 1 or 2 TC, N (%) +	1 (0.2)	1 (0.5)	0 (0)
Distance to the accepting TC, N (%)+	1 (0.2)	1 (0.5)	0 (0)
Time to the accepting TC, N (%)	49 (22.7)	35 (16.3)	14 (7.0)
Nearest TC level, N (%)+	1 (0.2)	1 (0.5)	0 (0)
Median household income, N (%)	3 (0.7)	2 (0.9)	1 (0.5)
Length of stay, N (%)	0 (0.0)	0 (0.0)	0 (0.0)
Death, N (%)	0 (0.0)	0 (0.0)	0 (0.0)
Hospital-free days at 90 days, N (%)	0 (0.0)	0 (0.0)	0 (0.0)

Variables included in multiple imputation: age, sex, injury type, ISS, mechanical ventilation, GCS, HR, SBP, transfer status, time to the accepting facility, death. Presence of SBP <90 and GCS <9 imputed passively from continuous variables.

+ For participants in whom trauma event location was missing, the centroid longitude and latitude of the forward sortation area of the home address was used as the presumed event location.

© 2024 Muttalib et al. *JAMA Netw. Open*.

eTable 5: Balance summary across all imputations

**Variable**	**Standardized difference between patients admitted to pediatric trauma centre vs. adult trauma centre**[+]
Propensity score	0.03
Sex	0.02
ISS	0.02
SBP <90	0.04
Mechanical ventilation	-0.0001
Injury type	
Asphyxia	0.008
Blunt	0.01
Other/Unspecified	0.04
Penetrating	0.02
GCS <9	0.04
Time to accepting facility	0.07

[+]Standardized difference less than 0.1 was considered to indicate acceptable covariate balance.[6]

© 2024 Muttalib et al. *JAMA Netw. Open*.

eFigure 2: Patients 12–16 years included in the primary analysis

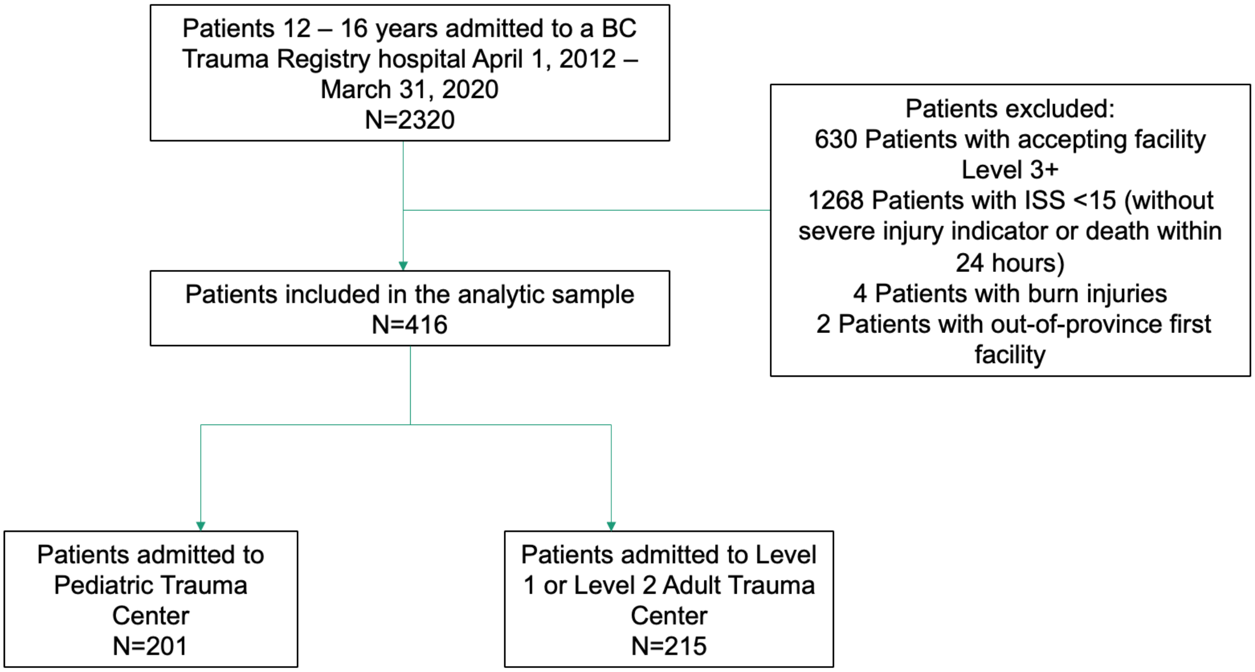

© 2024 Muttalib et al. *JAMA Netw. Open*.

eTable 6: Association between trauma centre type and mortality using logistic regression with inverse probability treatment weighting

**Predictors**	**Odds Ratios**	**Standard Error**	**CI**	**p**
(Intercept)	0.001	0.001	0.000 – 0.005	**<0.001**
Admission to PTC	2.61	1.44	0.88–7.69	**0.08**
Observations	416 (23 events)			

Abbreviations: PTC: Pediatric trauma centre, CI: Confidence interval
Variables included in the model: Injury severity score, trauma centre type

© 2024 Muttalib et al. *JAMA Netw. Open*.

eTable 7: Multivariable model for association between trauma centre type and hospital-free days at 90 days

	Incidence Rate Ratios	Std. Error	CI	P value
PTC [ref ATC]	1.02	0.02	0.99 – 1.06	0.20
Male Sex [ref Female]	1.02	0.02	0.98 – 1.06	0.34
ISS category [ref 1 – 8]				
9–14	0.97	0.03	0.92 – 1.02	0.19
15–24	0.96	0.03	0.92 – 1.02	0.16
25–34	0.88	0.03	0.83 – 0.94	**<0.001**
35–75	0.87	0.04	0.79 – 0.96	**0.006**
Presence of SBP <90	1.00	0.05	0.90 – 1.11	0.99
Presence of MV	0.95	0.03	0.90 – 0.99	**0.03**
Injury type [ref Blunt]				
Asphyxia	1.12	0.06	1.02-1.24	0.02
Penetrating	1.05	0.03	0.98-1.12	0.17
Unspecified/Other	1.03	0.12	0.83-1.29	0.77
GCS <9	0.98	0.04	0.91 – 1.06	0.63
Time to accepting facility	1.00	0.001	1.00 – 1.00	0.89

Abbreviations: ATC: Adult trauma centre, PTC: Pediatric trauma centre, ISS: injury severity score, SBP: Systolic blood pressure, MV: Mechanical ventilation, GCS: Glasgow coma scale, CI: Confidence interval

© 2024 Muttalib et al. *JAMA Netw. Open*.

eFigure 3: Patients 12–24 years included in the instrumental variable analysis

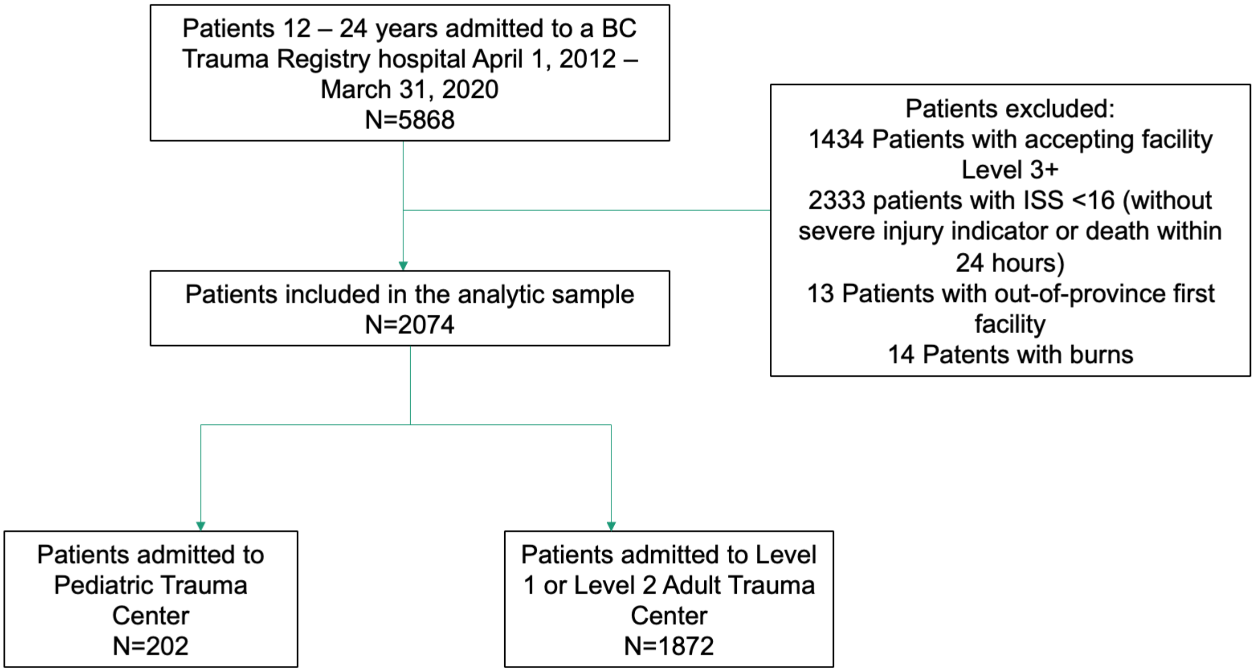

© 2024 Muttalib et al. *JAMA Netw. Open*.

eResults

Patient characteristics

Adolescents and young adults aged 12-24 years admitted to level 1 or 2 trauma centers
From April 1, 2012, to March 31, 2020, 2074 patients aged 12–24 years were admitted to a level 1 or level 2 trauma centre with severe injury, of whom 416 patients were 12–16 years (20%) and 1,658 patients were 17–24 years (80%) (eFigure 3). No patients were excluded due to missing age or outcome data. Patients admitted to adult trauma centres had injuries of greater severity, and a larger proportion were injured by a penetrating mechanism (eTable 7). Furthermore, patients transferred to an adult trauma centre had a shorter estimated distance to travel to reach their admitting hospital.
Most injuries were blunt (N=151 [75%] at the pediatric trauma centre, N=1,526 [82%] at adult trauma centres). Penetrating injuries were less common at the pediatric trauma centre (N=10, 5%) than adult trauma centres (N=281, 15%). Median ISS was lowest among patients admitted to the level 2 adult trauma centre 14 (IQR 9-22), versus pediatric trauma centre 16 (IQR 9-21) and the level 1 adult trauma centre 17 (IQR 10-26). A lower proportion of patients admitted to the pediatric trauma centre were direct admissions compared to patients admitted to adult trauma centres (N=73, 36% vs. N=1200, 64%).

Instrumental variable analysis
Examination of the instrument demonstrated that age category was a strong instrument for admission facility (F-test 1359.5, p<0.001). After adjusting for differences in baseline characteristics, instrumental variable analysis demonstrated that admission to pediatric trauma centre was associated with an average 5% increased absolute risk of hospital mortality (95%CI 0.2–9.1%, p=0.04) (eTable 9, eTable 10). Comparison of confounders by age category demonstrated that a greater proportion of patients aged 17–24 years had ISS 25–75 (20.5% among adolescents 12–16 yrs vs 28% among adults 17–24 yrs) and penetrating injuries (9.1% among patients 12-16 yrs vs 15% among patients 17–24 yrs). There were no other clinically meaningful differences. There was no significant association between risk of MVC and age category (p=0.31).

© 2024 Muttalib et al. *JAMA Netw. Open*.

eTable 8: Characteristics of adolescents and young adults 12–24 years hospitalized due to severe injury

	N	Overall, N = 2,074[a]	Level 1 PTC N = 202[a]	Level 1 or 2 ATC N = 1,872[a]
Age (years), median (IQR)	2,074	20 (17-22)	14 (13-15)	20 (18-22)
Sex, n (%)				
Male		1567 (75.6)	151 (74.8)	1416 (75.6)
Female	2,074	507 (24.4)	51 (25.2)	456 (24.4)
Injury type, n (%)	2,074			
Blunt		1,701 (82.0)	175 (86.6)	1,526 (81.5)
Penetrating		291 (14.0)	10 (5.0)	281 (15)
Other/Unspecified		82 (3.9)	17 (8.4)	65 (3.5)
ISS, median (IQR)	2,074	17 (10-25)	16 (9-21)	17 (10-25)
ISS category, n (%)	2,074			
1–8		259 (12.5)	45 (22.3)	214 (11.4)
9–15		653 (31.5)	48 (23.8)	605 (32.3)
16–24		617 (29.7)	65 (32.2)	552 (29.5)
25–75		545 (26.3)	44 (21.7)	501 (26.8)
Mechanical ventilation, n (%)	2,074	703 (33.9)	63 (31.2)	640 (34.2)
Heart Rate (bpm), median (IQR)	2,017	90 (76-105)	94 (80-106)	90 (76-105)
SBP, median (IQR)	2,015	125 (114-138)	118 (110-127)	126 (115-139)
Shock index, median (IQR)	1,991	0.72 (0.59-0.88)	0.78 (0.66-0.92)	0.71 (0.59-0.87)
SBP<90, n (%)	2,015	76 (3.8)	8 (4.3)	68 (3.7)
GCS<9, n (%)	1,885	267 (14.2)	26 (15.8)	241 (14.0)
First facility level, n (%)	2,074			
1 ATC		751 (36.2)	13 (6.4)	738 (39.4)
1 PTC		75 (3.6)	73 (36.1)	2 (0.1)
2		513 (24.7)	5 (2.5)	508 (27.1)
3		119 (5.7)	23 (11.4)	96 (5.1)
4		105 (5.1)	23 (11.4)	82 (4.4)
Other		511 (24.6)	65 (32.2)	446 (23.8)
Direct admission, n (%)	2,074	1,273 (61.4)	73 (35.9)	1,200 (64.1)
Accepting facility level, n (%)	2,074			
1 ATC		1,138 (54.9)	0 (0)	1,138 (60.8)
1 PTC		202 (9.7)	202 (100)	0 (0)
2		734 (35.4)	0 (0)	734 (39.2)
Minimum distance to level 1 or 2 TC (km), median (IQR)	2,070	14 (5-66)	23 (7-68)	13 (5,-64)

© 2024 Muttalib et al. *JAMA Netw. Open*.

Distance to the admitting TC (km), median (IQR)	2,070	18 (7-87)	40 (10-98)	17 (7-82)
Time to the accepting facility (hours), median (IQR)	1,804	2 (1-7)	5 (2-8)	1 (1-6)
Nearest TC level, n (%)	2,060			
1 ATC		414 (20.1)	53 (26.2)	361 (19.4)
1 PTC		215 (10.4)	40 (19.8)	175 (9.4)
2		1,431 (69.5)	109 (54.0)	1,322 (71.2)
Median neighbourhood household income (CAD), median (IQR)	2,052	69,922 (62,21-79,829)	70,212 (62,756-82,530)	69,818 (61,977-79,829)

[a] Median (IQR); n (%) Abbreviations: ATC: Adult trauma centre, PTC: Pediatric trauma centre, ISS: injury severity score, SBP: Systolic blood pressure, GCS: Glasgow coma scale, TC: Trauma centre, IQR: Interquartile range, CI: Confidence interval

© 2024 Muttalib et al. *JAMA Netw. Open*.

eTable 9: Multivariable model for association between trauma centre type and mortality among severely injured adolescents and adults using instrumental variable regression

	Risk difference	Standard error	95% CI	P value
PTC [ref ATC]	0.05	0.02	0.002 – 0.09	0.04
ISS category [ref 1 – 8]				
9–14	0.006	0.006	-0.005 – 0.02	0.3
15–24	0.007	0.005	-0.004 – 0.02	0.2
25–34	0.03	0.01	0.01 – 0.06	0.003
35–75	0.25	0.04	0.18 – 0.32	<0.001
Injury type [ref Blunt]				
Asphyxia	0.14	0.07	0.004 – 0.28	0.04
Penetrating	0.02	0.01	0.00002 – 0.04	0.22
Unspecified/Other	0.03	0.03	-0.02 – 0.09	0.05
Presence of SBP <90	0.14	0.05	0.05 – 0.23	0.003
Presence of MV	-0.02	0.01	-0.04 – 0.004	0.12
GCS < 9	0.17	0.03	0.12 – 0.21	<0.001
First facility TC Level 1 or 2 [ref 3,4 or non-TC]	-0.02	0.03	-0.07 – 0.03	0.5
Time to accepting facility	0.00002	0.00006	-0.0001 – 0.0001	0.93
Transfer [ref direct admission]	-0.02	0.03	-0.06 – 0.03	0.5

Abbreviations: ATC: Adult trauma centre, PTC: Pediatric trauma centre, ISS: injury severity score, SBP: Systolic blood pressure, MV: Mechanical ventilation, GCS: Glasgow coma scale, TC: Trauma centre, CI: Confidence interval

© 2024 Muttalib et al. *JAMA Netw. Open*.

eTable 10: Comparison of IV estimation methods of the average effect of PTC admission on hospital mortality

IV Estimation method	Risk difference (95% CI)	P value
Two stage least squares	0.05 (0.002 – 0.09)	0.04
Generalized method of moments	0.05 (0.002 – 0.09)	0.04
First stage probit, second stage OLS with fitted probabilities	0.05 (0.004 – 0.09)	0.03

Abbreviations: PTC: Pediatric trauma centre, OLS: Ordinary Least Squares regression

© 2024 Muttalib et al. *JAMA Netw. Open*.

eReferences

1. Hernan MA, Robins JM. Instruments for causal inference: an epidemiologist's dream? Epidemiology 2006;17:360-72.
2. Uddin MJG, R.H.; Ton De Boer,; Belitser, S.V.; Roes, K.C.; and Klungel, O.H. Instrumental Variable Analysis in Epidemiologic Studies: An Overview of the Estimation Methods. Pharmaceutica Analytica Acta 2015;6.
3. Wooldridge JM. Econometric Analysis of Cross Sectional and Panel Data. 2nd Edition ed. Cambridge, MA: MIT press; 2010.
4. Davies NM, Smith GD, Windmeijer F, Martin RM. Issues in the reporting and conduct of instrumental variable studies: a systematic review. Epidemiology 2013;24:363-369
5. Rassen JA, Schneeweiss S, Glynn RJ, Mittleman MA, Brookhart MA. Instrumental variable analysis for estimation of treatment effects with dichotomous outcomes. Am J Epidemiol 2009;169:273-84.
6. Austin PC, Stuart EA. Moving towards best practice when using inverse probability of treatment weighting (IPTW) using the propensity score to estimate causal treatment effects in observational studies. Stat Med 2015;34:3661-79.

© 2024 Muttalib et al. *JAMA Netw. Open*.
