# Supplementary material for: Mortality Among Severely Injured Adolescents Admitted to Pediatric vs Adult Trauma Centers
Source: JAMA Netw Open. 2024 Dec 12;7(12):e2450647. doi: 10.1001/jamanetworkopen.2024.50647 (PMC11638793; doi:10.1001/jamanetworkopen.2024.50647)
Supplement: Supplement 2. — Data Sharing Statement [file jamanetwopen-e2450647-s002.pdf]

Data Sharing Statement

Muttalib. Mortality Among Severely Injured Adolescents Admitted to Pediatric vs Adult Trauma Centers. *JAMA Netw Open*. Published December 12, 2024.
doi:10.1001/jamanetworkopen.2024.50647

Data
Data available: No

Additional Information
Explanation for why data not available: Data is available upon request to the BC Trauma Registry.
